# Different Temperature Regimes Affect Gonadal Development and Energy Budget in *Apostichopus japonicus*

**DOI:** 10.3390/ani16182815

**Published:** 2026-09-08

**Authors:** Jingzhao Zhang, Kaiyou Deng, Xueyu Zhu, Weijun Wang, Yanwei Feng, Xiaohui Xu, Zan Li, Cuiju Cui, Jianmin Yang, Guohua Sun

**Affiliations:** School of Fisheries, Ludong University, Yantai 264025, China; 18863872307@163.com (J.Z.); 17348990651@163.com (K.D.); 15735439008@163.com (X.Z.); wwj2530616@163.com (W.W.); fywzxm1228@163.com (Y.F.); xxh83121@163.com (X.X.); lizanlxm@163.com (Z.L.); cuicuiju@163.com (C.C.); ladderup@126.com (J.Y.)

**Keywords:** stepwise warming, thermal biology, gametogenesis, energy allocation, reproductive physiology, broodstock conditioning

## Abstract

*Apostichopus japonicus* is a temperate marine echinoderm whose growth and reproduction are strongly influenced by temperature. We compared adult sea cucumbers exposed to stepwise warming (10–12, 12–14, and 14–16 °C across three successive 20-day stages) with animals maintained at 15 °C. Growth indices, gonadosomatic index (GSI), gonadal histology, and energy-budget components were evaluated in females and males. The 60 d experiment used three replicate tanks for each treatment-by-sex combination. Compared with the constant 15 °C treatment, the stepwise-warming regime showed a higher female GSI at stage III; representative gonadal sections documented stage-related structural changes in both treatments. No significant treatment effects were detected for final wet weight, specific growth rate, daily growth rate, or feed conversion ratio. Excretory energy decreased during stages II and III, while the proportion allocated to gonadal growth increased. Stage-specific within-sex comparisons showed higher gonadal growth energy under stepwise warming in both sexes during stage II, while the clearest GSI difference occurred in females during stage III; treatment × sex interactions were not significant. The clearest differences between the two temperature regimes were observed during stages II and III (12–16 °C). The findings provide an integrative physiological basis for temperature management during broodstock conditioning of *A. japonicus*.

## Simple Summary

Water temperature is one of the main environmental signals controlling reproduction in the Japanese sea cucumber. Most laboratory studies use a fixed temperature, although animals in nature experience gradual seasonal warming. We compared adults maintained at 15 °C with adults exposed to a three-stage warming treatment from 10 to 16 °C. We examined body growth, gonad structure, and how food-derived energy was divided among losses, maintenance, body growth, and gonad growth. Representative gonadal sections from both temperature treatments showed stage-related structural progression during stages II and III, while specific and daily growth rates were generally maintained. Energy lost through excretion declined, and a larger share of energy was associated with gonad growth. The clearest difference in gonad weight relative to body weight between treatments was detected in females at stage III. These findings improve understanding of how seasonal temperature patterns coordinate growth and reproduction in a marine invertebrate and may help refine temperature management during sea cucumber broodstock conditioning.

## 1. Introduction

*Apostichopus japonicus* (Selenka, 1867) is a temperate benthic marine animal endemic to the western North Pacific and has high edible and medicinal value [1]. To meet continuously increasing market demand, artificial aquaculture of *A. japonicus* has expanded rapidly in China and has become one of the major pillars of marine aquaculture [1,2]. In recent years, sea cucumber farming has continued to expand and has shown strong regional clustering. According to the China Fishery Statistical Yearbook 2025, national sea cucumber aquaculture production in 2024 reached 326,172 tons, with a farming area of 268,467 ha [3]. The *A. japonicus* industry therefore occupies an important position in China’s marine aquaculture economy. A stable supply of healthy juveniles is the foundation for sustainable development of this industry. However, in artificial breeding systems, key technical steps during broodstock conditioning, including standardized maturation induction and regulation of individual developmental variation, still suffer from insufficient theoretical support. This leads to poor technical stability, efficiency, and reproducibility and restricts quality improvement, efficiency enhancement, and sustainable development of the industry [2,4].

*A. japonicus* is a typical dioecious marine invertebrate. Its gonadal development and reproduction show clear seasonality and environmental dependence, with water temperature being especially important. Temperature is a core ecological factor regulating gonadal development and reproduction in this species. On the one hand, temperature indirectly controls gamete maturation and release by affecting metabolism and endocrine regulation, including neurohormonal control [5,6]. Recent experimental work in *Holothuria arguinensis* also showed that temperature and photoperiod jointly influence gametogenesis, highlighting the importance of seasonal environmental cues in sea cucumbers [7]. On the other hand, an appropriate temperature range directly determines the occurrence of reproductive activity. Previous studies show that *A. japonicus* usually enters the spawning period at 15–23 °C, with an optimal spawning temperature of approximately 18–20 °C [8,9]. Within this range, gonadal development reaches its peak, and sperm and eggs can be released synchronously. Based on histological and morphological features, the reproductive cycle of *A. japonicus* is usually divided into five consecutive stages: the post-spawning resting or recovery stage, the proliferative stage marked by the initiation of germ-cell proliferation, the mature stage with peak gonadosomatic index (GSI), the spawning stage characterized by gamete release, and the post-spawning stage featuring gonadal regression [8]. Under natural conditions, the reproductive cycle usually follows an annual rhythm. This annual cycle and its clear stage structure are strongly driven by temperature. Suitable temperatures of 15–23 °C promote gonadal maturation and spawning, and this temperature-reproduction coupling provides an important theoretical basis for artificial seed production and germplasm regulation in *A. japonicus*. Accordingly, the 10–16 °C trajectory used in this study was intended to represent the progressive warming phase preceding the principal spawning-temperature range.

Bioenergetic analysis provides an important theoretical framework for studying how organisms acquire, transform, allocate, and use energy. It is widely used to explain growth, reproduction, environmental adaptation, and population ecology in aquatic animals, and it also supports optimization of aquaculture systems, precise feeding management, and improvement of farming efficiency [10]. Bioenergetic models provide a unified framework for quantifying energy acquisition, transformation, allocation, and expenditure. The core relationship is expressed as the energy budget equation C = S + U + R + G [10,11], in which consumed energy is allocated to growth, respiration, fecal loss, and excretion. This model is widely used in ecological energetics of aquaculture animals to reveal how energy is distributed among maintenance, growth, immunity, and reproduction under different environmental conditions. It is also applied to assessments of carrying capacity, feed-efficiency optimization, and ecological risk prediction under climate change [12,13,14]. For *A. japonicus*, energy metabolism is closely coupled with environmental conditions. Previous studies show that temperature is an important environmental factor affecting energy metabolism in *A. japonicus*. Recent multi-omics evidence further shows that low-temperature stress is accompanied by changes in oxidative metabolism, the tricarboxylic acid (TCA) cycle, oxidative phosphorylation, and lipid and amino acid metabolism in *A. japonicus* [15]. It regulates feeding, metabolism, and growth and further affects the allocation of energy among maintenance, growth, and reproduction [10,11]. In addition, salinity, feeding ratio, feeding frequency, and stocking density can also change the energy budget and physiological performance of *A. japonicus* [10,16,17].

Nevertheless, most bioenergetic studies on *A. japonicus* and other aquaculture animals have been conducted under constant-temperature conditions. In natural sea areas and in most farming environments, water temperature usually shows diel, seasonal, and gradual fluctuations rather than remaining stable at a single constant value for a long period [18]. Compared with constant temperature, fluctuating temperature often requires individuals to invest additional energy in maintaining ionic homeostasis, cellular repair, and antioxidant regulation, thereby changing the allocation of energy among maintenance, growth, and storage [19,20]. Recent work in *A. japonicus* also showed that temperature and salinity stress can alter respiratory carbon fluxes and metabolizable energy [21]. Thus, physiological parameters and energy-budget results obtained only under constant-temperature conditions may not accurately reflect the growth performance and ecological adaptation of *A. japonicus* under natural or practical aquaculture conditions. Energy-budget studies under dynamic temperature conditions are therefore important for understanding the physiological response mechanisms of *A. japonicus* to environmental change, improving sea cucumber bioenergetic models, and optimizing temperature-control strategies in production.

Temperature change not only affects gonadal development but may also alter reproductive strategy by regulating energy acquisition, expenditure, and allocation. Reproductive investment is also linked to nutritional and metabolic status; in broodstock fish, dietary phospholipid supply has been shown to affect GSI, sex steroid production, and tissue fatty-acid profiles [22]. Clarifying the energy-allocation mechanism underlying temperature-driven growth and reproduction in *A. japonicus* is therefore important for optimizing broodstock conditioning, improving the stability of juvenile production, and supporting sustainable aquaculture development. Although previous studies have shown that temperature affects feeding, metabolism, and growth in *A. japonicus*, how this species adjusts its energy-allocation pattern during seasonal stepwise warming remains insufficiently understood. In particular, systematic evidence is still lacking on how energy is partitioned between somatic growth (Gs) and gonadal growth (Gg) during the transition from early gonadal development to maturation. Therefore, this study simulates a natural seasonal warming process by establishing a stepwise-warming treatment and a constant-temperature control. We hypothesized that stepwise warming would reduce energy losses and increase the proportion of energy allocated to gonadal development, with different response timing between females and males. We systematically evaluate growth performance, gonadal development, and dynamic energy-budget responses in adult *A. japonicus*, with a particular focus on the allocation of energy between somatic growth and gonadal development. The findings provide new insight into how temperature variation and sex differences shape energy allocation and reproductive investment in *A. japonicus* and offer a theoretical basis for precise temperature management during broodstock conditioning.

## 2. Materials and Methods

### 2.1. Animals and Experimental Design

Adult *Apostichopus japonicus* used in this experiment were obtained from Yantai Shenyuanda Seed Industry Technology Co., Ltd., Yantai, Shandong Province, China. Before the experiment, healthy individuals with uniform size and early-stage gonadal development were selected. Their initial mean wet weight was 275.66 ± 59.32 g (mean ± standard deviation [SD]). *A. japonicus* has been reported to reach sexual maturity at approximately 3 years of age, with estimated sizes at sexual maturity of 81.71 g for females and 83.94 g for males [23]. All individuals were first acclimated for 7 d in a 720 L glass tank (150 cm × 60 cm × 80 cm) to adapt to laboratory culture conditions. Before experimental allocation, sex was preliminarily identified by experienced technicians at the source hatchery based on broodstock-breeding experience and external morphological characteristics; females and males were then maintained separately in the corresponding experimental tanks. A total of 120 individuals were randomly assigned to 12 independent glass tanks with an effective water volume of 56.6 L (38 cm × 39 cm × 38 cm), with 10 same-sex individuals in each tank. The experiment included a stepwise-warming treatment and a constant-temperature control, with three replicate tanks for each treatment-by-sex combination (six tanks per temperature treatment in total).

The experiment was conducted from 20 April to 18 June 2025 (60 d), and each 20 d period was defined as one observation stage. The control group was maintained at a constant temperature of 15 °C throughout the experiment. The treatment group was exposed to stepwise warming with reference to the natural seasonal warming process. This trajectory was selected to represent the early seasonal warming phase associated with gonadal development before the main spawning-temperature range. Water temperature gradually increased from approximately 10–12 °C in stage I to approximately 12–14 °C in stage II and then to approximately 14–16 °C in stage III. Stages I, II, and III corresponded to days 1–20, 21–40, and 41–60, respectively. The primary aim of this study was not to compare responses at several discrete constant-temperature points, but to simulate the physiological process of *A. japonicus* under seasonal stepwise warming. These temperature ranges characterized the warming level of each stage rather than requiring water temperature to remain strictly fixed at a single value at every time point. Water temperature in each tank was regulated using heating equipment and recorded once in the morning and once in the evening each day to ensure that the treatment group followed a stable stage-specific warming trend. Based on the twice-daily records, mean temperatures in the constant-temperature treatment were 15.06 ± 0.12, 15.06 ± 0.18, and 15.11 ± 0.13 °C during stages I–III, respectively. Corresponding values in the stepwise-warming treatment were 11.17 ± 0.63 °C (10.1–12.0 °C), 12.97 ± 0.69 °C (12.0–14.0 °C), and 15.01 ± 0.66 °C (14.0–16.2 °C), with approximate warming rates of 0.10, 0.12, and 0.11 °C d^−1^. The complete 60 d water-temperature profiles for both regimes are provided in Supplementary Figure S1.

During the experiment, all *A. japonicus* were fed once daily at 10:00 with a commercial pellet diet (Weihai Jinpai Feed Co., Ltd., Weihai, China) at 1% of the total wet weight in each tank. To ensure accurate feeding amounts, individuals in each tank were weighed at the end of each 20 d stage, and daily feeding amounts for the next stage were adjusted according to the new total wet weight. All experimental tanks were maintained under the same culture conditions. Approximately one-third of the tank water was replaced daily with filtered natural seawater, and continuous aeration was provided to maintain water quality. Key water-quality parameters were maintained at pH 8.86–9.27, salinity 29.58–29.81‰, and dissolved oxygen 5.79–6.62 mg/L.

### 2.2. Sample Collection and Measurement

Before the experiment, three females and three males were randomly sampled from the same batch as initial samples. Thereafter, at the end of each stage, one individual was randomly sampled from each of the three replicate tanks for every treatment-by-sex combination, yielding three females and three males from each temperature treatment as terminal samples for that stage. Tank means were used as the experimental units in statistical analysis. After sampling, individual wet weight was measured. The gonads and remaining tissues excluding the gonads were then dissected. Tissue samples were dried at 70 °C to constant mass, ground into homogeneous powder, and stored at −80 °C for subsequent body-composition and energy measurements. At the same time, dry feed offered to each tank was recorded. Before the next feeding, uneaten cylindrical pellets and feces were recovered; because they were readily distinguishable in gross morphology, they were separated using silk-mesh filtration and forceps. Recovered uneaten feed and feces were dried separately at 70 °C to constant mass and weighed. Actual feed intake was calculated as dry feed offered minus dry recovered uneaten feed, and fecal samples were stored at −80 °C for subsequent calorific-value measurement. To estimate excretory energy, seawater samples were collected from the upper, middle, and lower layers of each tank before and after each water exchange and then mixed. Each sample volume was 50 mL, and samples from the three replicate tanks were analyzed for each treatment and sex. Samples were frozen at −80 °C until analysis.

Total nitrogen (TN) concentrations in seawater were determined using a potassium persulfate oxidation method. After alkaline thermal oxidation, nitrate was reduced through a cadmium–copper column and quantified spectrophotometrically after diazotization and azo coupling at 540 nm; TN was expressed as mg L^−1^. Gross calorific values of feed, feces, gonads, and nongonadal tissues were determined on a dry-matter basis by oxygen-bomb calorimetry using benzoic acid for calibration.

### 2.3. Histological Preparation and Descriptive Examination of Gonads

To document gonadal structure at the different experimental stages, gonadal tissues were collected from female and male *Apostichopus japonicus* at the end of each stage and processed for paraffin sectioning and hematoxylin and eosin (H&E) staining. After fixation, tissue samples were dehydrated through a graded ethanol series, cleared in xylene, infiltrated and embedded in paraffin, and cut into serial sections using standard histological procedures. Sections were deparaffinized, rehydrated, stained, dehydrated, cleared, mounted, observed, and photographed under a light microscope. The sections were examined descriptively for visible germ-cell morphology and arrangement, cytoplasmic staining, follicular or tubular organization, and luminal occupation. Quantitative morphometry and standardized developmental scoring were not performed. Because the displayed fields were selected to illustrate representative morphology rather than through a prespecified stereological sampling scheme, they were not used to derive post hoc treatment-level estimates of cell size, cell number, or occupied area.

### 2.4. Growth and Energy Indices

*Apostichopus japonicus* in each tank were weighed and counted at the end of each stage to calculate growth indices and energy-budget parameters. Growth-related indices include final wet weight (Mw), which is the wet weight at the end of each experimental stage and is used to evaluate growth status; specific growth rate (SGR), which measures the relative increase in body mass per unit time; daily growth rate (DGR), which measures the average daily growth per unit time; and feed conversion ratio (FCR), which represents feed intake per unit of weight gain and is used to evaluate feed-use efficiency. A lower FCR indicates higher feed conversion efficiency. In these calculations, Wi is initial body mass, Wf is final body mass, and t is feeding time. These growth indices are commonly used in *A. japonicus* growth and energy-budget studies [16,17]. The gonadosomatic index (GSI) was calculated as gonad wet weight relative to total individual wet body weight [8], with Mw representing total individual wet body weight at sampling and Gw representing gonad wet weight. Previous reproductive studies have also used gonadal indices to characterize reproductive status [24].

SGR (%) = 100 × (ln Wf − ln Wi)/t

DGR (%) = 100 × (Wf − Wi)/(Wi × t)

FCR = feed intake (dry weight)/weight gain (g)

GSI (%) = Gw/Mw × 100

The energy budget is calculated as C = S + U + R + G, where G = Gs + Gg and A = G + R = Gs + Gg + R. Because all energy-budget calculations in this study are based on dried samples, subsequent energy conversions uniformly use the gross calorific value on a dry basis (Qgr,d). Here, C is energy consumption, S is fecal energy, U is excretory energy, R is metabolic energy, G is total growth energy, Gs is somatic growth energy excluding gonads but including the digestive tract, respiratory tree, other visceral tissues, and body wall, Gg is gonadal growth energy, and A is assimilated energy. In this study, C, S, and G were derived from measured feed, fecal, and tissue energies. Excretory energy was estimated as U (kcal) = 24.83 [(TN_old − TN_new) × V × T]/4184, where TN_old and TN_new are total nitrogen concentrations before and after water exchange (mg L^−1^), V is the effective water volume (L), T is the time interval (d), 24.83 J mg^−1^ N is the energetic equivalent of nitrogen excretion, and 1 kcal = 4184 J. Metabolic energy R was estimated as the residual of the energy-budget equation: R = C − G − S − U [10,17,25].

### 2.5. Statistical Analysis

All results were expressed as mean ± SD. Statistical analyses were performed using SPSS 26.0 (IBM Corp., Armonk, NY, USA). For each experimental stage, growth and energy-budget variables were analyzed by two-way analysis of variance (ANOVA), with temperature treatment (constant 15 °C vs. stepwise warming) and sex (female vs. male) as fixed factors and the treatment × sex interaction included in the model. The replicate tank was treated as the experimental unit (n = 3 per treatment × sex combination). When treatment comparisons within each sex were required, planned simple-effect comparisons were performed using the residual error term from the corresponding ANOVA with Bonferroni adjustment. Model residuals were examined for normality using the Shapiro–Wilk test and homogeneity of variance was evaluated using Levene’s test. Test statistics, degrees of freedom, and exact *p* values are reported for inferential results; *p* < 0.05 was considered significant. The three stages were analyzed separately because each represented a predefined 20 d experimental interval and, in the stepwise-warming treatment, was intrinsically associated with a different absolute temperature range. Consequently, an overall treatment × stage interaction would combine elapsed time, developmental progression, and absolute-temperature differences and could not be interpreted as an independent temperature-trajectory effect. Given the three replicate tanks per treatment × sex combination, nonsignificant interaction effects were interpreted cautiously.

## 3. Results

### 3.1. Growth Performance

**Figure 1:**
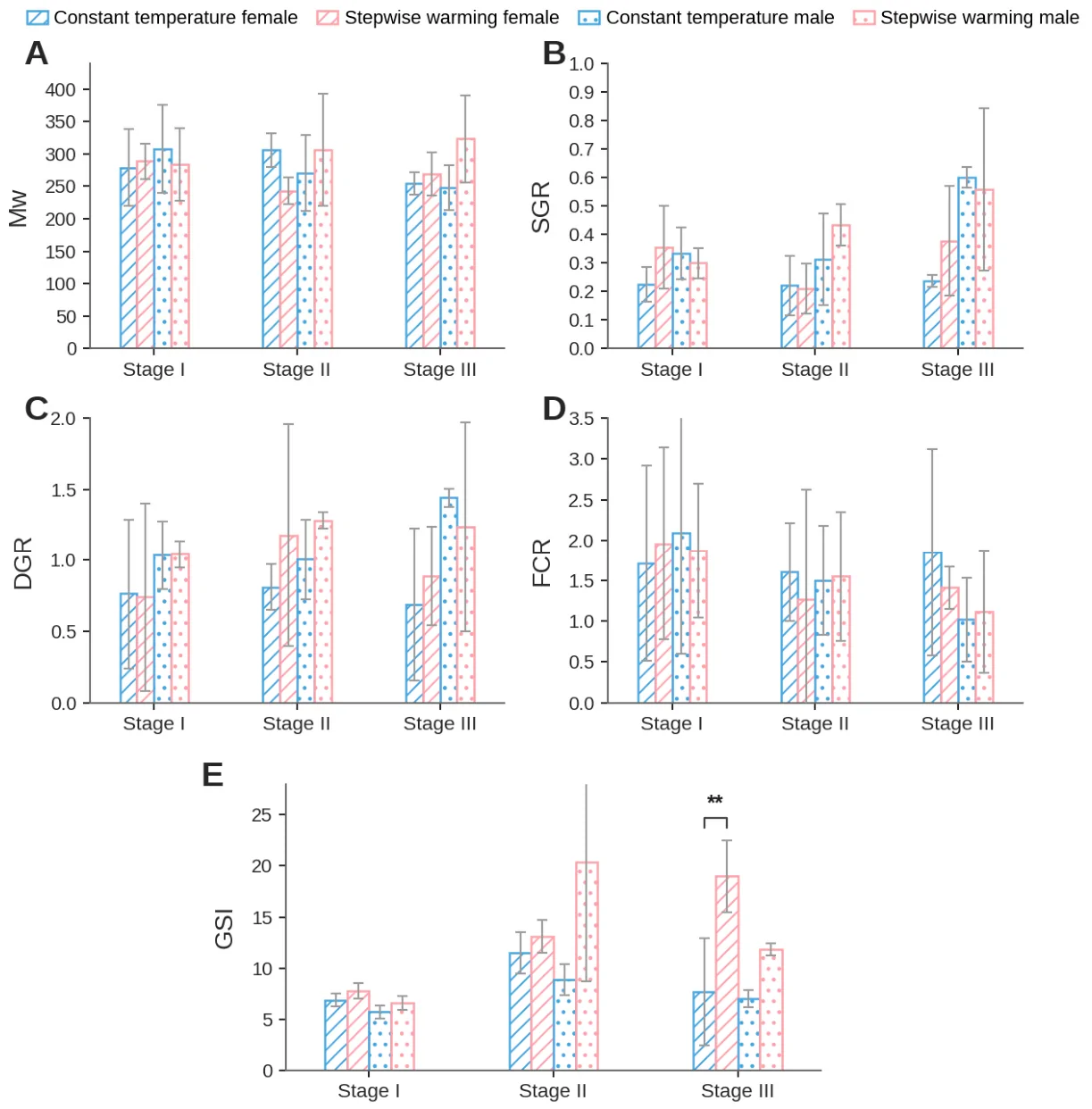
Changes in growth indices and gonadosomatic index of female and male *Apostichopus japonicus* at different experimental stages under different temperature treatments. (**A**) Final wet weight (Mw); (**B**) specific growth rate (SGR); (**C**) daily growth rate (DGR); (**D**) feed conversion ratio (FCR); (**E**) gonadosomatic index (GSI). Stage I, stage II, and stage III indicate the first, second, and third experimental stages, respectively. Blue bars with diagonal lines indicate females in the constant-temperature group, red bars with diagonal lines indicate females in the stepwise-warming group, blue dotted bars indicate males in the constant-temperature group, and red dotted bars indicate males in the stepwise-warming group. Double asterisks indicate a Bonferroni-adjusted planned comparison between the temperature treatments within the same stage and sex (** *p* < 0.01).

Growth indices and GSI across the three experimental stages are shown in Figure 1. During the 60 d experiment, the survival rate of *Apostichopus japonicus* was 100% in all treatment and control groups. No significant treatment × sex interaction was detected for Mw, SGR, DGR, FCR, or GSI at any stage (all *p* ≥ 0.116). At stage I, GSI showed a significant sex effect (*F*(1, 8) = 8.929, *p* = 0.017), whereas the treatment effect did not reach significance (*F*(1, 8) = 5.027, *p* = 0.055); no treatment comparison within either sex was significant after Bonferroni adjustment. At stage II, SGR showed a significant sex effect (*F*(1, 8) = 6.094, *p* = 0.039), while no planned treatment comparison remained significant after adjustment. At stage III, SGR again showed a significant sex effect (*F*(1, 8) = 7.455, *p* = 0.026), and temperature treatment had a significant effect on GSI (*F*(1, 8) = 19.133, *p* = 0.002). Female GSI was higher under stepwise warming than under constant 15 °C (19.00 ± 3.51 vs. 7.68 ± 5.25; *t*(8) = 4.339, adjusted *p* = 0.005), whereas the corresponding male comparison was not significant (11.81 ± 0.62 vs. 6.99 ± 0.82; *t*(8) = 1.847, adjusted *p* = 0.204). No significant treatment main effects were detected for Mw, SGR, DGR, or FCR at any stage.

### 3.2. Representative Histological Features of Female and Male Gonads

**Figure 2:**
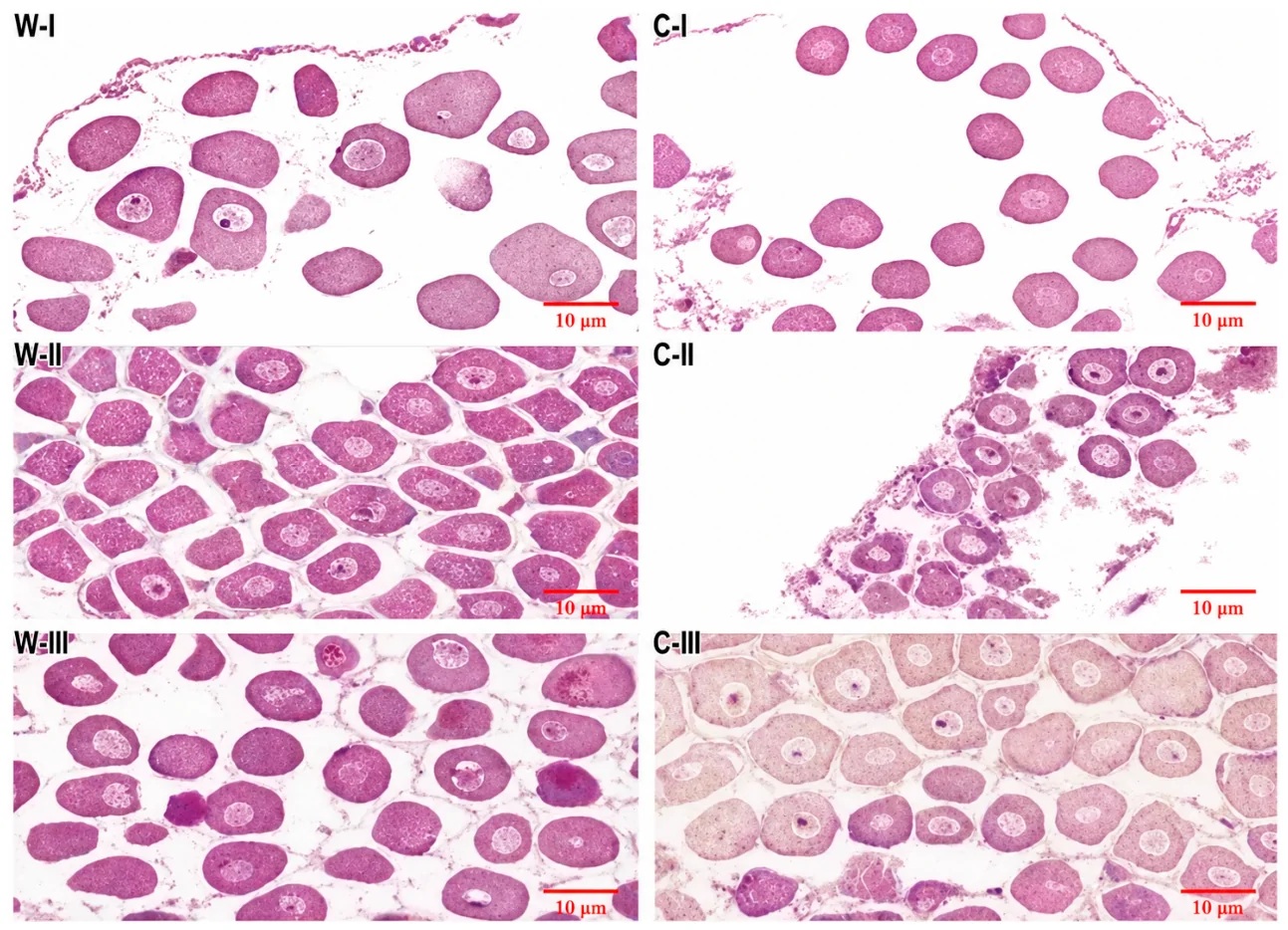
Representative hematoxylin and eosin (H&E)-stained sections of female *Apostichopus japonicus* gonads at different experimental stages under different temperature treatments. W-I, W-II, and W-III indicate stages I, II, and III in the stepwise-warming group, respectively. C-I, C-II, and C-III indicate stages I, II, and III in the constant-temperature group, respectively. Scale bars = 10 μm.

**Figure 3:**
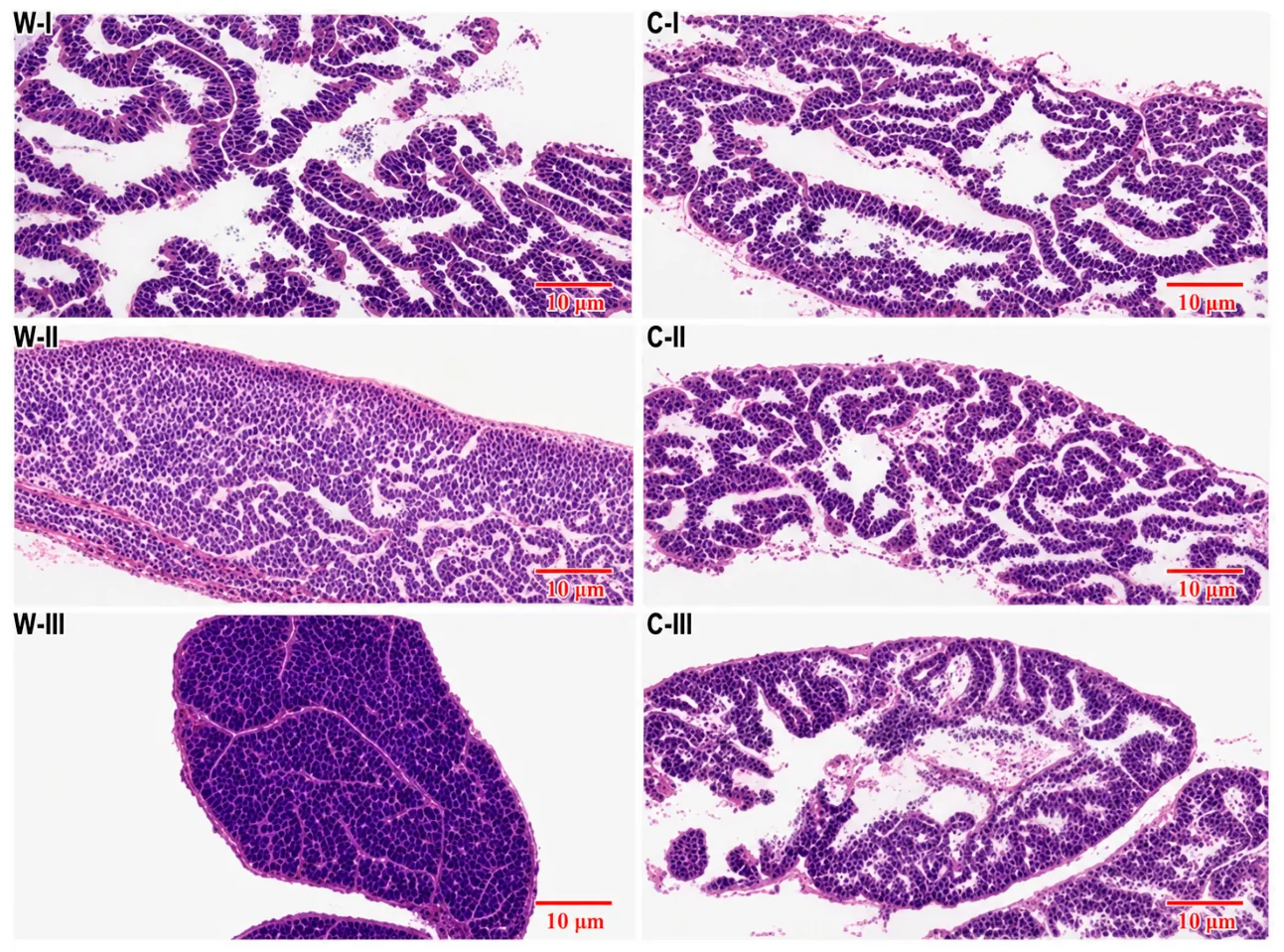
Representative hematoxylin and eosin (H&E)-stained sections of male *Apostichopus japonicus* gonads at different experimental stages under different temperature treatments. W-I, W-II, and W-III indicate stages I, II, and III in the stepwise-warming group, respectively. C-I, C-II, and C-III indicate stages I, II, and III in the constant-temperature group, respectively. Scale bars = 10 μm.

Representative female and male sections from stage I showed early developmental features in both temperature treatments. The female sections (W-I and C-I in Figure 2) contained relatively sparse oocyte profiles with open intercellular spaces and no conspicuous cytoplasmic granule accumulation in the displayed fields. The male sections (W-I and C-I in Figure 3) showed gonadal tubules with substantial open luminal space and no uniformly dense sperm-filled region.

At stage II, the displayed female sections contained more closely arranged oocyte profiles than at stage I, indicating continued structural development in both treatments. The W-II field showed densely packed oocyte profiles, whereas the C-II field showed a more heterogeneous distribution (Figure 2). In males, the W-II field showed densely arranged spermatogenic cells and reduced open luminal space relative to W-I, while the C-II field also showed increased cellular occupation relative to C-I (Figure 3).

At stage III, representative female sections from both treatments contained numerous well-developed oocyte profiles and reduced intercellular space. The displayed W-III and C-III fields differed in local cellular arrangement, but both showed continued gonadal development (Figure 2). The W-III male field showed dense cellular occupation of the gonadal tubules, whereas the displayed C-III field retained larger open luminal regions (Figure 3). These representative sections document visible structural features only; no treatment-level morphometric or standardized scoring inference was made from the selected fields.

### 3.3. Energy-Budget Analysis

**Figure 4:**
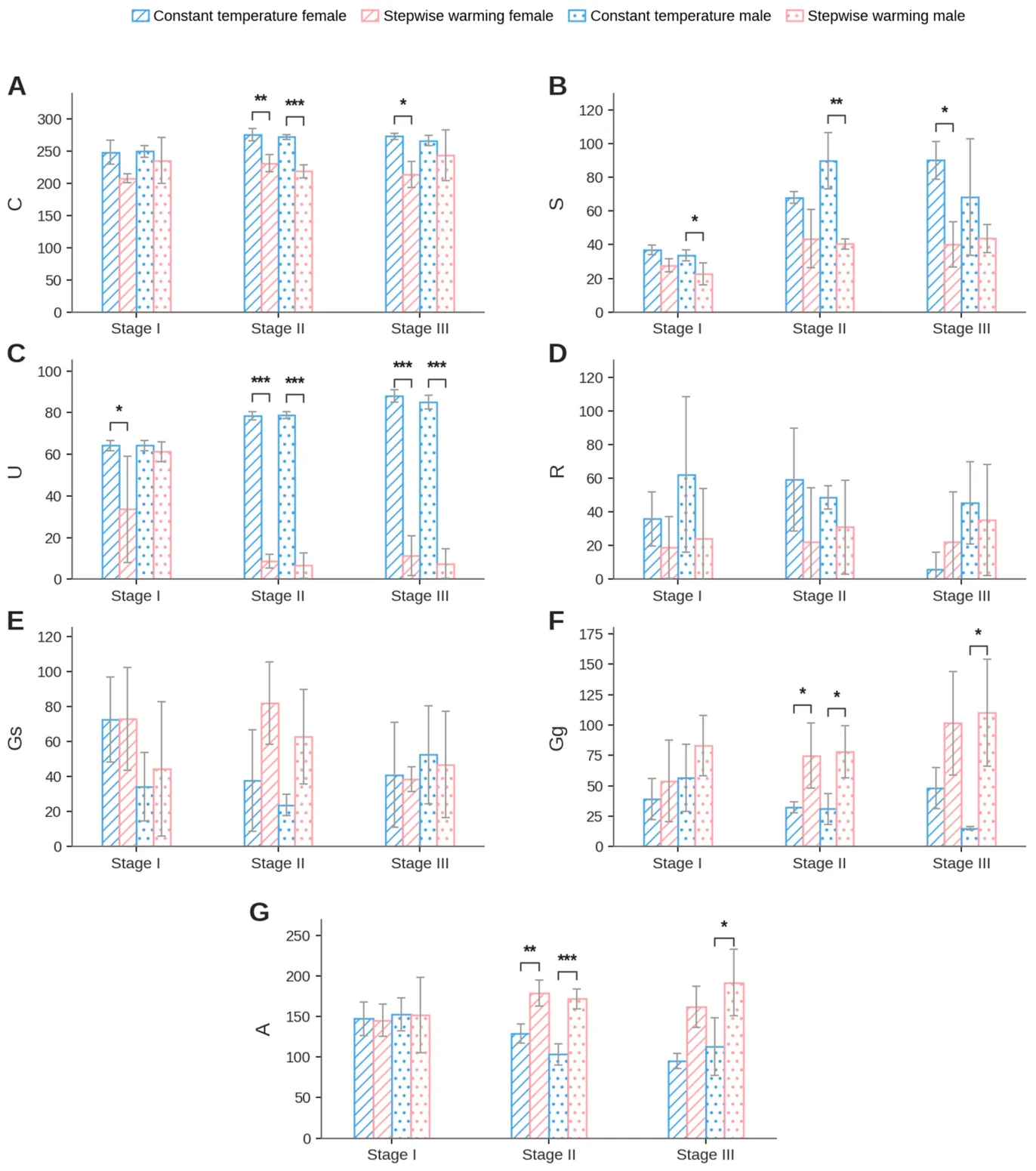
Changes in energy-budget components of female and male *Apostichopus japonicus* at each experimental stage under different temperature treatments. (**A**) Energy consumption (C); (**B**) fecal energy (S); (**C**) excretory energy (U); (**D**) metabolic energy (R); (**E**) somatic growth energy (Gs); (**F**) gonadal growth energy (Gg); (**G**) assimilated energy (A). Stage I, stage II, and stage III correspond to 10–12 °C, 12–14 °C, and 14–16 °C in the stepwise-warming treatment, respectively, whereas the constant-temperature control was maintained at 15 °C throughout the experiment. Blue bars with diagonal lines indicate females in the constant-temperature group, red bars with diagonal lines indicate females in the warming group, blue dotted bars indicate males in the constant-temperature group, and red dotted bars indicate males in the warming group. Asterisks indicate Bonferroni-adjusted planned comparisons between temperature treatments within the same stage and sex (* *p* < 0.05; ** *p* < 0.01; *** *p* < 0.001). The y-axis unit of each index is kcal.

Changes in energy-budget components are shown in Figure 4, and the corresponding absolute energy-budget indices and energy-allocation ratios are summarized in Table 1 and Table 2, respectively. No significant treatment × sex interaction was detected for any energy-budget variable at stage I (all *p* > 0.05). Fecal energy (S) showed a significant treatment effect (*F*(1, 8) = 15.621, *p* = 0.004), whereas the treatment effects for energy consumption (C; *F*(1, 8) = 4.930, *p* = 0.057) and excretory energy (U; *F*(1, 8) = 4.900, *p* = 0.058) did not reach significance. A sex effect was detected for Gs/C (*F*(1, 8) = 5.982, *p* = 0.040). Planned comparisons showed lower S in warming-group males than in constant-temperature males (*t*(8) = −3.015, adjusted *p* = 0.033) and lower U in warming-group females (*t*(8) = −2.854, adjusted *p* = 0.043). No other adjusted within-sex treatment comparison was significant at stage I.

At stage II, treatment effects were significant for C (*F*(1, 8) = 75.861, *p* < 0.001), S (*F*(1, 8) = 27.162, *p* < 0.001), U (*F*(1, 8) = 1148.331, *p* < 0.001), somatic growth energy Gs (*F*(1, 8) = 9.686, *p* = 0.014), gonadal growth energy Gg (*F*(1, 8) = 17.639, *p* = 0.003), and assimilated energy A (*F*(1, 8) = 59.313, *p* < 0.001), whereas R was not significantly affected by treatment (*F*(1, 8) = 3.221, *p* = 0.110). Treatment effects were also significant for S/C (*F*(1, 8) = 15.714, *p* = 0.004), U/C (*F*(1, 8) = 570.746, *p* < 0.001), Gs/C (*F*(1, 8) = 16.293, *p* = 0.004), Gg/C (*F*(1, 8) = 25.335, *p* = 0.001), G/A (*F*(1, 8) = 8.817, *p* = 0.018), and R/A (*F*(1, 8) = 8.817, *p* = 0.018). In females, C (*t*(8) = −5.611, adjusted *p* = 0.001) and U (*t*(8) = −23.558, adjusted *p* < 0.001) were lower, whereas Gg (*t*(8) = 2.827, adjusted *p* = 0.045) and A (*t*(8) = 4.594, adjusted *p* = 0.004) were higher. Female U/C decreased (*t*(8) = −16.497, adjusted *p* < 0.001), while Gs/C (*t*(8) = 2.974, adjusted *p* = 0.036) and Gg/C (*t*(8) = 3.293, adjusted *p* = 0.022) increased. In males, C (*t*(8) = −6.707, adjusted *p* < 0.001), S (*t*(8) = −4.926, adjusted *p* = 0.002), and U (*t*(8) = −24.366, adjusted *p* < 0.001) were lower, whereas Gg (*t*(8) = 3.112, adjusted *p* = 0.029) and A (*t*(8) = 6.297, adjusted *p* < 0.001) were higher. Male S/C (*t*(8) = −3.962, adjusted *p* = 0.008) and U/C (*t*(8) = −17.289, adjusted *p* < 0.001) decreased and Gg/C increased (*t*(8) = 3.825, adjusted *p* = 0.010). Treatment × sex interactions were not significant for these variables (all *p* > 0.05).

At stage III, treatment effects were significant for C (*F*(1, 8) = 9.905, *p* = 0.014), S (*F*(1, 8) = 10.562, *p* = 0.012), U (*F*(1, 8) = 446.273, *p* < 0.001), Gg (*F*(1, 8) = 16.399, *p* = 0.004), A (*F*(1, 8) = 17.191, *p* = 0.003), U/C (*F*(1, 8) = 322.949, *p* < 0.001), and Gg/C (*F*(1, 8) = 30.419, *p* < 0.001). In females, C (*t*(8) = −3.201, adjusted *p* = 0.025), S (*t*(8) = −3.078, adjusted *p* = 0.030), U (*t*(8) = −14.845, adjusted *p* < 0.001), and U/C (*t*(8) = −12.239, adjusted *p* < 0.001) were lower, whereas Gg/C was higher (*t*(8) = 3.362, adjusted *p* = 0.020). In males, U (*t*(8) = −15.030, adjusted *p* < 0.001) and U/C (*t*(8) = −13.175, adjusted *p* < 0.001) were lower, whereas Gg (*t*(8) = 3.668, adjusted *p* = 0.013), A (*t*(8) = 3.177, adjusted *p* = 0.026), and Gg/C (*t*(8) = 4.438, adjusted *p* = 0.004) were higher. Treatment × sex interactions were not significant (all *p* > 0.05).

## 4. Discussion

### 4.1. Temperature-Regime Differences in Gonadal Development of Apostichopus japonicus

Representative gonadal sections documented stage-related structural changes in both temperature regimes. The displayed later-stage sections showed dense oocyte packing or spermatogenic-cell occupation in selected fields, but the histological material was examined descriptively and does not provide a quantitative treatment comparison. The quantified GSI analysis showed a significant treatment effect at stage III, with the planned within-sex difference detected in females. Together with the energy-budget results, these findings indicate that the clearest measured reproductive differences between the two regimes occurred during the later experimental stages, while the histological images provide morphological context rather than independent statistical confirmation.

This phenomenon is not unique to *A. japonicus*. Temperature change within a specific range, especially stepwise or gradual warming, is a core environmental cue that regulates reproductive rhythm and promotes gonadal development and maturation. Recent experimental work in the sea cucumber *Holothuria arguinensis* showed that photoperiod strongly influenced gametogenesis and that temperature had a modulatory role [7]. Temperature also modulates reproductive performance in the Chinese mitten crab *Eriocheir sinensis*; reproductive performance and embryonic development vary across 6–21 °C, with optimal reproductive performance around 15 °C [26]. In bivalves, temperature changes are also important factors inducing gonadal maturation and regulating reproductive processes. In the tropical mussel *Perna perna*, temperature and food ration jointly affect gametogenesis and growth, indicating that adequate nutritional supply under suitable thermal conditions can promote reproductive development in bivalves [27]. Similar patterns also occur in echinoderms, which are phylogenetically closer to *A. japonicus*. In the sea urchin *Paracentrotus lividus*, 18–22 °C promotes gonadal development, and 20–22 °C supports higher gonadal growth and maturity [28]. A large body of evidence confirms that gonadal development in aquaculture animals depends on specific water-temperature ranges and thermal accumulation. Each step from gonadal initiation and differentiation to maturation and final reproduction is highly sensitive to changes in water temperature and has its own suitable thermal window [29,30]. For *A. japonicus*, gonadal development under natural conditions enters a rapid growth stage when water temperature rises to approximately 13–16 °C [8,9]. Broodstock conditioning and spawning of this species are also strongly influenced by temperature management [9]. In addition, *A. japonicus* shows an income-breeding strategy, in which reproductive investment relies heavily on energy acquired and used during the breeding period [31]. From an applied perspective, the response of *A. japonicus* to the tested warming regime provides information relevant to broodstock conditioning. Because the present experiment compared a progressive 10–16 °C regime with a constant 15 °C treatment, the effect of thermal trajectory cannot be separated fully from that of absolute temperature at each stage. The 12–16 °C interval should therefore be interpreted as the later portion of the tested warming regime rather than as a uniquely defined thermal window. By integrating a dynamic temperature trajectory with sex-specific histology, growth indices, and energy-budget analysis, the present study extends previous constant-temperature research on *A. japonicus*.

### 4.2. Gonadal Development, Maintenance of Somatic Growth Homeostasis, and Internal Energy Reallocation

Throughout the culture period, no significant treatment effect was detected for Mw, SGR, DGR, or FCR at any stage (all *p* > 0.05). Thus, the measured somatic growth indices remained comparable between the two temperature regimes. Similar phenomena have been reported in other aquaculture animals. In European sea bass, *Dicentrarchus labrax*, water temperature can affect gonadal differentiation while also influencing growth, suggesting that reproductive development does not necessarily inhibit individual growth [32]. In echinoderms, *P. lividus* can achieve favorable gonadal growth and sexual maturation under certain temperature conditions [28]. In *A. japonicus*, temperature physiology studies show that growth has a clear suitable temperature window. Small or moderate temperature fluctuations do not suppress growth and may instead improve growth and physiological performance [33]. Within suitable low-temperature ranges, *A. japonicus* can also maintain high feeding activity, feed conversion efficiency, and specific growth rate [16]. These findings indicate that when temperature variation remains within an appropriate range, *A. japonicus* can maintain its basic capacity for somatic growth and provide the physiological basis needed for reproductive development. In the present study, the later stages of the stepwise-warming regime were accompanied by greater gonadal growth energy in several comparisons without significant treatment effects on the measured somatic growth indices.

From an energy-budget perspective, the combination of higher gonadal growth energy at later stages, a higher female GSI at stage III, and generally stable somatic growth in this study more likely reflects reorganization of internal energy allocation rather than simple growth suppression. In other aquaculture animals, temperature change often reshapes energy acquisition, expenditure, and allocation, thereby altering internal energy balance. In *Lateolabrax maculatus*, temperature significantly affects ovarian development and reveals different energy-metabolism configurations between reproductive tissues and muscle tissues [34]. In echinoderms, studies on *Paracentrotus lividus* show that energy budget and resource allocation patterns change markedly under different temperature conditions [35]. In *Psammechinus miliaris*, temperature not only participates in the regulation of gametogenesis but also affects the energy allocation relationship between gonadal growth and body growth [36]. For *A. japonicus*, Xie et al. [10] systematically measured energy-budget parameters under different water temperatures, including energy consumption, fecal energy, excretory energy, metabolic energy, growth energy, and growth potential. Their results show that water temperature affects not only energy acquisition and expenditure but also the energy available for growth. Ru et al. [25] further report, in a study of energy-budget adjustment during the reproductive period of *A. japonicus*, that individuals reduce part of their energy loss, lower the proportions of feeding and loss, and increase the proportion of energy allocated to reproduction as gonadal growth progresses. In addition, the dynamic energy budget (DEB) model constructed and parameterized for *A. japonicus* by Ren et al. [11] indicates that ingested energy can be allocated among maintenance metabolism, growth, reproduction, and other physiological processes, and that environmental change can further alter energy allocation among physiological functions by affecting energy acquisition, assimilation, and expenditure. The present results are consistent with this theoretical framework. During stages II and III, the stepwise-warming group showed lower U and U/C together with higher gonadal growth energy (Gg) and Gg/C in several comparisons, indicating a shift in measured energy allocation between the two temperature regimes. Because R was calculated as the residual term of the energy-budget equation, it should be interpreted as a budget estimate rather than a direct measurement of metabolic rate.

### 4.3. Sex-Related Patterns in Reproductive Development and Energy Allocation

Treatment × sex interactions were not significant for the analyzed growth or energy-budget variables. Nevertheless, planned within-sex comparisons showed stage-specific patterns. At stage II, gonadal growth energy (Gg) was higher under the stepwise-warming regime in both females and males (adjusted *p* = 0.045 and 0.029, respectively). At stage III, the treatment difference in GSI was significant in females (adjusted *p* = 0.005) but not in males (adjusted *p* = 0.204), whereas male Gg and A were higher under the stepwise-warming regime (adjusted *p* = 0.013 and 0.026, respectively). These results identify stage-specific within-sex differences but do not demonstrate statistically different treatment sensitivity between females and males. Because only three replicate tanks were available per treatment × sex combination, nonsignificant interactions should be interpreted cautiously.

Differences in developmental degree and energy allocation between sexes are not unique to *A. japonicus*. They are also common in other aquatic animals, although the direction of the difference is strongly species specific. In fish, an annual reproductive-cycle study of *Nibea albiflora* shows that males can release sperm in both spring and autumn, whereas females show a continuous spawning period from spring to autumn. Seasonal peaks of the early germ-cell marker gene *vasa* in the testis and ovary are not synchronized, suggesting different temporal windows for reproductive initiation and activity between sexes [37]. Studies on early gonadal differentiation in *Siniperca chuatsi* also show that female differentiation mainly occurs at 27–51 days post-fertilization (dpf), whereas male differentiation mainly occurs at 33–57 dpf, indicating that the progression of gonadal development can differ markedly between sexes even within the same species [38]. Such sex-specific developmental asynchrony is often accompanied by different energy-use and reproductive-investment strategies. For example, wild European plaice show significant sex-based differences in annual energy use, with males having lower energy expenditure than females in summer and the opposite pattern outside summer [39]. In *Labrus bergylta*, females and males differ in how they mobilize stored energy, reflecting distinct reproductive investment strategies [40]. Similar patterns also occur in echinoderms. In the brooding sea urchin *Abatus cavernosus*, male gonadal growth and gonopore development show inflection points at smaller body sizes, and males reach maturity-related stages earlier than females, indicating clear sex-specific developmental asynchrony in echinoderms [41]. For *A. japonicus*, although direct comparative studies on the seasonal timing of developmental initiation in males and females remain limited, existing evidence indicates that males and females differ in reproductive energy use. Female *A. japonicus* can reduce locomotor activity during reproduction to maintain energy homeostasis and meet higher reproductive-investment demands [42]. In addition, male and female *A. japonicus* differ significantly in intestinal and gonadal lipid metabolism, and these differences are considered to be associated with distinct reproductive investment modes and physiological demands [43]. Recent field evidence further indicates that sexual maturity in *A. japonicus* is associated with body-energy-reserve thresholds of approximately 105.49 kJ in females and 109.78 kJ in males [23]. Gamete maturation and spawning behavior are also important components of reproductive investment; relaxin-like gonad-stimulating peptide has been shown to promote gamete maturation and spawning in *A. japonicus* [6]. These observations provide biological context for the stage-specific patterns detected here, although the treatment × sex interactions were not significant.

Sex-related differences in lipid and reproductive physiology have been reported in aquatic animals and may provide context for the patterns observed here. In echinoderms, the fatty acid composition of male and female gonads differs markedly in *P. lividus* and *Arbacia lixula*, suggesting that oogenesis and spermatogenesis require different substrate types and metabolic pathways [44]. The gonadal transcriptome of *P. lividus* further identifies many sex-related and lipid-biosynthesis-related differentially expressed genes [45]. In *A. japonicus*, Zhang et al. [43] compared lipid metabolism in intestinal and gonadal tissues between males and females and found significant sex differences in gonadal lipid metabolism. In addition, gonadal transcriptome studies in *A. japonicus* identify multiple sex-biased cytochrome P450 (*CYP*) genes and microRNA (miRNA)–messenger RNA (mRNA) regulatory networks involved in gonadal differentiation and reproductive regulation [46,47]. Stage-specific expression of follicle-stimulating hormone β-subunit (*FSHβ*) and luteinizing hormone β-subunit (*LHβ*) has also been reported during gonadal development in giant gourami [48], providing comparative evidence that endocrine regulation can vary across reproductive stages in aquatic animals. However, endocrine signaling, lipid mobilization, and vitellogenesis were not measured in the present experiment, so these mechanisms are not inferred from our data.

## 5. Conclusions

This study simulates a natural seasonal stepwise warming process and systematically evaluates gonadal development, somatic growth, and energy-budget responses in adult *Apostichopus japonicus* under different temperature regimes. Compared with constant culture at 15 °C, individuals exposed to the 10–16 °C stepwise-warming regime showed a higher female GSI at stage III and stage-related structural changes in representative gonadal sections, without significant treatment effects on Mw, SGR, DGR, or FCR. During stages II and III, the two temperature regimes differed markedly in energy allocation, with lower U and U/C and higher gonadal growth energy and Gg/C detected in several comparisons under the stepwise-warming regime. At stage III, GSI showed a significant treatment effect, and the within-sex difference was detected in females. Although females and males showed different stage-specific patterns, treatment × sex interactions were not significant. Accordingly, the 12–16 °C interval is interpreted as the later portion of the tested warming regime rather than as a uniquely defined thermal window. These findings provide physiological information on gonadal development and energy allocation under contrasting temperature regimes and may help refine temperature management during *A. japonicus* broodstock conditioning. Future studies should combine analyses of respiratory metabolism, endocrine regulation, terminal reproductive traits, and energy-sensing pathways to further clarify the mechanisms underlying temperature-related energy reallocation and gonadal maturation.

## Figures and Tables

**Table 1:** Absolute energy-budget indices of *Apostichopus japonicus* at different experimental stages under different temperature treatments.

Stage	Sex	Treatment	C (kcal)	S (kcal)	U (kcal)	R (kcal)	Gs (kcal)	Gg (kcal)	A (kcal)
I	Female	Constant temperature	248.34 ± 18.71	36.92 ± 3.02	64.19 ± 2.58	35.98 ± 16.08	72.52 ± 24.40	38.91 ± 17.01	147.42 ± 20.95
		Stepwise warming	207.89 ± 7.38	27.63 ± 3.91	33.65 ± 25.50	18.78 ± 18.35	72.98 ± 29.27	53.86 ± 33.55	145.62 ± 19.75
	Male	Constant temperature	249.56 ± 9.14	33.52 ± 3.24	64.19 ± 2.58	62.24 ± 46.18	34.20 ± 19.58	56.40 ± 27.64	152.84 ± 20.09
		Stepwise warming	235.66 ± 36.17	22.63 ± 6.59	61.22 ± 4.86	24.24 ± 29.81	44.45 ± 38.37	83.16 ± 24.80	151.85 ± 46.86
II	Female	Constant temperature	275.57 ± 9.17	67.96 ± 3.42	78.59 ± 2.03	59.25 ± 30.62	37.56 ± 29.19	32.21 ± 4.38	129.02 ± 11.93
		Stepwise warming	231.17 ± 13.34	43.51 ± 17.34	8.76 ± 3.25	22.12 ± 32.23	81.94 ± 23.57	74.88 ± 26.95	178.94 ± 15.93
	Male	Constant temperature	272.03 ± 3.66	89.73 ± 16.71	78.84 ± 1.51	48.76 ± 6.86	23.80 ± 5.93	30.91 ± 12.91	103.47 ± 13.11
		Stepwise warming	218.97 ± 10.01	40.45 ± 3.00	6.61 ± 5.98	31.04 ± 27.92	62.97 ± 27.29	77.88 ± 21.32	171.89 ± 11.76
III	Female	Constant temperature	273.11 ± 4.47	90.23 ± 11.20	88.10 ± 3.00	5.86 ± 9.97	41.05 ± 29.93	48.32 ± 16.90	95.23 ± 9.71
		Stepwise warming	213.90 ± 19.96	40.31 ± 13.40	11.40 ± 9.46	22.07 ± 30.52	38.43 ± 6.98	101.70 ± 42.62	162.19 ± 25.67
	Male	Constant temperature	266.45 ± 8.20	68.26 ± 34.65	85.17 ± 3.33	45.34 ± 24.39	52.59 ± 28.16	15.06 ± 1.39	113.00 ± 35.92
		Stepwise warming	243.34 ± 39.59	43.65 ± 8.51	7.51 ± 7.11	35.17 ± 33.08	46.87 ± 29.87	110.15 ± 43.91	192.19 ± 41.67

Note: Values are presented as mean ± standard deviation (SD) (*n* = 3 replicate tanks per treatment × sex combination). C, energy consumption; S, fecal energy; U, excretory energy; R, metabolic energy; Gs, somatic growth energy; Gg, gonadal growth energy; A, assimilated energy.

**Table 2:** Energy allocation ratios of *Apostichopus japonicus* at different experimental stages under different temperature treatments.

Stage	Sex	Treatment	S/C	U/C	Gs/C	Gg/C	R/C	G/A	R/A
I	Female	Constant temperature	0.15 ± 0.02	0.26 ± 0.03	0.29 ± 0.10	0.16 ± 0.08	0.14 ± 0.05	0.76 ± 0.07	0.24 ± 0.07
		Stepwise warming	0.13 ± 0.02	0.16 ± 0.12	0.35 ± 0.14	0.26 ± 0.17	0.09 ± 0.09	0.88 ± 0.11	0.12 ± 0.11
	Male	Constant temperature	0.13 ± 0.02	0.26 ± 0.03	0.14 ± 0.08	0.23 ± 0.11	0.25 ± 0.18	0.61 ± 0.29	0.39 ± 0.29
		Stepwise warming	0.10 ± 0.04	0.27 ± 0.05	0.18 ± 0.13	0.35 ± 0.05	0.11 ± 0.14	0.82 ± 0.23	0.18 ± 0.23
II	Female	Constant temperature	0.25 ± 0.02	0.29 ± 0.02	0.14 ± 0.11	0.12 ± 0.01	0.21 ± 0.11	0.54 ± 0.22	0.46 ± 0.22
		Stepwise warming	0.19 ± 0.07	0.04 ± 0.02	0.35 ± 0.08	0.32 ± 0.11	0.10 ± 0.15	0.87 ± 0.20	0.13 ± 0.20
	Male	Constant temperature	0.33 ± 0.06	0.29 ± 0.01	0.09 ± 0.02	0.11 ± 0.05	0.18 ± 0.02	0.52 ± 0.11	0.48 ± 0.11
		Stepwise warming	0.18 ± 0.01	0.03 ± 0.03	0.28 ± 0.11	0.35 ± 0.09	0.15 ± 0.14	0.81 ± 0.17	0.19 ± 0.17
III	Female	Constant temperature	0.33 ± 0.04	0.32 ± 0.01	0.15 ± 0.11	0.18 ± 0.07	0.02 ± 0.04	0.93 ± 0.11	0.07 ± 0.11
		Stepwise warming	0.19 ± 0.08	0.05 ± 0.04	0.18 ± 0.03	0.47 ± 0.16	0.11 ± 0.15	0.85 ± 0.20	0.15 ± 0.20
	Male	Constant temperature	0.26 ± 0.13	0.29 ± 0.06	0.20 ± 0.11	0.06 ± 0.00	0.17 ± 0.09	0.59 ± 0.21	0.41 ± 0.21
		Stepwise warming	0.19 ± 0.07	0.03 ± 0.03	0.18 ± 0.10	0.44 ± 0.12	0.16 ± 0.18	0.78 ± 0.25	0.22 ± 0.25

Note: Values are presented as mean ± standard deviation (SD) (*n* = 3 replicate tanks per treatment × sex combination). S/C, fecal energy/energy consumption; U/C, excretory energy/energy consumption; Gs/C, somatic growth energy/energy consumption; Gg/C, gonadal growth energy/energy consumption; R/C, metabolic energy/energy consumption; G/A, total growth energy/assimilated energy; R/A, metabolic energy/assimilated energy.

## Data Availability

The raw data supporting the conclusions of this article are available from the corresponding author upon reasonable request.

## Supplementary Materials

The following supporting information can be downloaded at https://www.mdpi.com/article/10.3390/ani16182815/s1. Figure S1: Water-temperature profiles during the 60 d experiment under the constant-temperature and stepwise-warming regimes.

## Abbreviations

The following abbreviations are used in this manuscript:

A	assimilated energy
C	energy consumption
DGR	daily growth rate
FCR	feed conversion ratio
G	total growth energy
Gs	somatic growth energy
Gg	gonadal growth energy
GSI	gonadosomatic index
H&E	hematoxylin and eosin
Mw	final wet weight
R	metabolic energy
S	fecal energy
SGR	specific growth rate
U	excretory energy
